# Parental Burnout and Adolescents’ Growth Mindset: An Exploratory Two-Wave Study

**DOI:** 10.3390/bs16071194

**Published:** 2026-07-15

**Authors:** Dayu Zhao, Jia Luo, Jianqiang Wang, Wei Wang, Yongxin Li

**Affiliations:** Institute of Psychology and Behaviour, Henan University, Kaifeng 475001, China

**Keywords:** parental burnout, parental neglect, parental violence, growth mindset, cross-lagged panel model

## Abstract

Purpose: Owing to rapid economic development and increasing social complexity, parenting stress has intensified for many families. When parents are exposed to parenting-related stress without sufficient coping resources, they may become vulnerable to parental burnout. Based on two-wave parent–adolescent dyadic data, this study explored whether parental neglect and parental violence may serve as potential indirect pathways linking parental burnout to adolescents’ growth mindset. Methods: Using a semi-longitudinal design, we collected two waves of survey data one month apart from 382 middle-school students and their parents in a city in Central China. An initial cross-lagged panel model (CLPM) was estimated based on the proposed hypotheses. Because the initial model showed poor fit, it was modified based on the modification indices and theoretical considerations, yielding the final model. Results: The final model showed acceptable fit, χ^2^(15) = 39.15, RMSEA = 0.065, CFI = 0.978, and TLI = 0.952. T1 parental burnout was significantly and positively associated with T2 neglect (β = 0.20, *p* < 0.001), and T1 neglect was significantly and negatively associated with T2 growth mindset (β = −0.13, *p* < 0.01). The indirect effect = −0.02, 95% CI [−0.033, −0.003], *p* < 0.05. Supplementary analyses also showed a generally consistent pattern of results. Conclusions: These findings provide preliminary evidence that parental neglect may be an important linking pathway between parental burnout and lower levels of adolescents’ growth mindset.

## 1. Introduction

Rapid economic development and increasing social diversification have created growing pressures on parents related to work, finances, broader social environments, and parenting expectations. These evolving demands have further intensified the burdens associated with childrearing. According to the Balance between Risks and Resources model (Mikolajczak & Roskam, 2018), parental burnout may occur when parenting resources (e.g., self-compassion, high emotional competence, effective parenting practices, leisure time, positive co-parenting, and external support) are chronically outweighed by parenting-related risks (e.g., parental perfectionism, low emotional competence, dysfunctional parenting, excessive household workload, and insufficient social support). Parental burnout refers to a syndrome that emerges when parents experience a prolonged imbalance between parenting demands and available resources. It is characterized by parents feeling exhausted in their parenting role, detaching themselves emotionally from their children, not enjoying being with their children, losing a sense of fulfillment in parenting, and not recognizing themselves as the parents they want to be (Roskam et al., 2018).

Existing research has shown that parental burnout exerts negative effects on parents’ psychological functioning and behaviors. It increases the risk of depression, anxiety, sleep disturbances, substance use, and tendencies toward escape and suicidal ideation (Mikolajczak et al., 2019). It may also lead to regret about having children (Piotrowski et al., 2025). At the couple level, parental burnout intensifies marital conflict and decreases marital satisfaction (W. Wang et al., 2022). Moreover, because parenting is inherently a bidirectional interaction between parents and adolescents, parents’ psychological states and behavioral patterns ultimately affect their offspring through everyday parenting practices. Previous studies have indicated that parental burnout may result in poor mental health, compromised social adjustment, decreased sense of security, and increased problem behaviors among adolescents (Guo et al., 2024; Song et al., 2024; B. Yang et al., 2021). Recent person-centered research further found that a higher-risk group of parents, characterized by elevated parental burnout and parenthood regret, along with impaired parental reflective functioning, was associated with higher levels of child dysregulation (Carone et al., 2025). Additionally, parents who experience chronic parental burnout tend to have lower-quality parent–child relationships, which leave their offspring feeling isolated and unsupported at school and in social settings, thereby making it difficult for them to build stable peer relationships and ultimately hindering the development of their social adjustment abilities (Gillis & Roskam, 2019).

These findings substantially deepen our understanding of the potential negative associations of parental burnout on adolescents’ healthy development. However, several important indicators of adolescents’ growth and development have yet to be sufficiently discussed or empirically examined. In this context, a growth mindset is an important psychological resource for adolescents’ learning beliefs and responses to academic setbacks (Aditomo, 2015). A growth mindset refers to the belief that abilities, intelligence, and potential are malleable and can be developed through effort, strategies, and experience. In contrast, a fixed mindset assumes that abilities are innate or unchangeable, which may make individuals more prone to feelings of helplessness and giving up when facing failure (Dweck & Yeager, 2019). A growth mindset can enhance individuals’ psychological resilience and emotion regulation, enabling them to show more adaptive responses when encountering setbacks or failures (Schroder et al., 2017). It is closely associated with psychological well-being because it decreases the likelihood of depression and anxiety symptoms among adolescents and improves their ability to cope with stressful situations (X. Yang et al., 2024). Conversely, individuals with a fixed mindset are more likely to attribute failure to their perceived lack of ability, which may lead to diminished self-esteem and increased negative emotions (Gál et al., 2022). In academic settings, such individuals are also prone to academic anxiety and avoidance of challenges (Tao et al., 2022). Moreover, adolescence is a critical period during which self-conceptions and beliefs about ability undergo rapid development, making the cultivation of a growth mindset particularly important (Park et al., 2020).

During the development of a growth mindset among adolescents, parents’ beliefs, feedback practices, and emotional support within family interactions may play an important role in shaping adolescents’ understanding of the malleability of their abilities (Gunderson et al., 2013). Parents experiencing burnout typically display emotional exhaustion and psychological distancing (Mikolajczak et al., 2018). In such contexts, parents may be less able to provide timely recognition and encouragement of their adolescents’ efforts and progress, which may make it more difficult for adolescents to perceive abilities as improvable through effort and learning (Muenks et al., 2017). Moreover, parental emotional support and positive feedback can effectively enhance adolescents’ growth mindset, thereby fostering greater resilience and motivation when facing challenges (Yeager & Dweck, 2012). At the same time, a reduced parental sense of achievement and a strong perceived contrast with the past self (e.g., parents believing they are no longer “good parents”), which are typical features of parental burnout, may also be linked to adolescents’ growth mindset. Specifically, if parents exhibit a strong fear of failure, adolescents may interpret failure as a reflection of their abilities rather than an opportunity for growth (Haimovitz & Dweck, 2016). Such negative responses to failure may promote the development of a fixed mindset, leading adolescents to believe that their abilities cannot be changed or improved.

Accordingly, we proposed Hypothesis 1.

**H1.**  *Parental burnout is negatively associated with adolescents’ growth mindset.*

Parental burnout has been shown to be associated with parental neglect and violence toward children (Mikolajczak & Roskam, 2018). This association has been consistently supported and examined across different types of data analyses, including cross-sectional (Mikolajczak & Roskam, 2018) and longitudinal studies (Schittek et al., 2024), individual-centered research (Hansotte et al., 2020), and studies conducted across diverse cultural contexts (Roskam et al., 2021). Parenting behaviors may be associated with adolescents’ mental health (Yap et al., 2014). For example, limited parental communication, attention, and involvement in adolescents’ school activities, learning, and everyday emotional experiences may reduce the support adolescents receive for learning and emotional adjustment and may be associated with poorer academic and emotional functioning (M.-T. Wang & Sheikh-Khalil, 2014). In addition, parental violence has been associated with higher levels of internalizing and externalizing problems among adolescents (Peltonen et al., 2010). Based on the above evidence, these negative parenting behaviors may also be associated with adolescents’ growth mindset by reducing emotional support, feedback, and opportunities for adaptive interpretations of effort and failure.

Taken together, parental neglect and parental violence may be relevant to the association between parental burnout and adolescents’ growth mindset. Therefore, we proposed the following exploratory indirect association hypotheses:

**H2a.**  *Neglect mediates the relationship between parental burnout and adolescents’ growth mindset.*

**H2b.**  *Violence mediates the relationship between parental burnout and adolescents’ growth mindset.*

## 2. Current Study

Most existing studies have used cross-sectional designs (Roskam & Mikolajczak, 2023) and relied mainly on parental self-reports (Roskam et al., 2017). To address these limitations, the present study adopted a semi-longitudinal design in which data on all variables were collected at two time points, and matched data were collected from parents and adolescents at the family level. This design allowed us to control for autoregressive paths to some extent and to examine the temporal ordering among the study variables. Parental burnout was reported by parents, whereas parental neglect, parental violence, and growth mindset were reported by adolescents. Because adolescents’ self-reports may more directly capture the neglect and violence they experience firsthand (De Los Reyes & Kazdin, 2005), this design allowed us to examine whether parental burnout was associated with adolescents’ growth mindset and whether parental neglect and parental violence may represent potential indirect pathways in this association.

## 3. Method

### Participants and Procedure

This study targeted middle-school students and their parents. Cluster sampling was employed to distribute questionnaires in a middle school located in a city in Central China. The survey included two versions: one for parents and one for adolescents. Parents completed a questionnaire assessing parental burnout, while adolescents reported on perceived parental neglect and violence, as well as their growth mindset. Adolescents completed and returned their questionnaires on site during a 30 min break. Parent questionnaires were brought home by adolescents in sealed envelopes; after completion, parents returned the sealed questionnaires to school through their adolescents, who then submitted them to the research team. A semi-longitudinal design was employed, with two waves of data collection conducted one month apart. The first wave was conducted in March 2022, when 400 parent–adolescent dyads were invited and 384 questionnaires were returned. The second wave was conducted one month later with the same cohort of 400 parent–adolescent dyads, and 384 questionnaires were returned. For matching purposes, adolescents were required to report their names, and parents were asked to report the names of their adolescents. All participants provided informed consent, and privacy was strictly maintained. Although names were collected for matching, they were not included in the subsequent analyses. After matching parent and adolescent questionnaires across the two waves, the analytic dataset included 382 parent–adolescent dyads with at least partial data on the study variables. In the parent sample, the mean age was 40.84 years (*SD* = 6.54), including 86 fathers with a mean age of 41.85 years (*SD* = 6.31). In the adolescent sample, the mean age was 13.04 years (*SD* = 0.74), including 202 boys with a mean age of 13.11 years (*SD* = 0.74).

The study involving human participants was reviewed and approved by the Ethics Committee of the authors’ institution (protocol code 20190329001).

## 4. Measures

### 4.1. Parental Burnout

Parental burnout was assessed using the simplified Chinese version of the Parental Burnout Assessment (W. Wang et al., 2021). The scale consists of seven items, such as “I can no longer endure being a parent.” Responses are rated on a 7-point Likert scale ranging from 0 (“completely disagree”) to 6 (“completely agree”), with higher scores indicating higher levels of parental burnout. In this study, the scale demonstrated good internal consistency, with Cronbach’s α = 0.893 at Time 1 and Cronbach’s α = 0.914 at Time 2.

### 4.2. Neglect

Parental neglect was assessed using the neglect subscale of the Child Psychological Maltreatment Scale (Pan et al., 2010). This scale has been validated in Chinese contexts (Li et al., 2022). The scale consists of six items, such as “My parents do not communicate with me about my school activities.” Responses are rated on a 5-point Likert scale ranging from 0 (“never”) to 4 (“always”), with higher scores indicating higher levels of neglect. In this study, the scale demonstrated good internal consistency, with Cronbach’s α = 0.852 at Time 1 and Cronbach’s α = 0.871 at Time 2.

### 4.3. Violence

Parental violence was assessed using a four-item harsh parenting measure (Simons et al., 1991). This measure has been validated in Chinese contexts (D. Wang et al., 2025). The scale includes four items, such as “When I do something wrong, my parent hits me with their hand or kicks me.” Responses are rated on a 5-point Likert scale ranging from 1 (“never”) to 5 (“always”), with higher scores indicating higher levels of harsh parenting experienced by the adolescent. In this study, the scale demonstrated good internal consistency, with Cronbach’s α = 0.829 at Time 1 and Cronbach’s α = 0.897 at Time 2.

### 4.4. Growth Mindset

Adolescents’ growth mindset was assessed using the Growth Mindset Scale (Dweck, 2013; De Castella & Byrne, 2015). This scale has been validated in Chinese contexts (Zhou et al., 2020). The scale consists of eight items across two dimensions: four items directly assess growth mindset, whereas the other four assess fixed mindset and are reverse-scored. An example item is, “No matter your level of intelligence, you can always make some changes.” Responses are rated on a 5-point Likert scale ranging from 1 (“strongly disagree”) to 5 (“strongly agree”), with higher scores indicating a stronger tendency toward a growth mindset. In this study, the scale demonstrated good internal consistency, with Cronbach’s α = 0.866 at Time 1 and Cronbach’s α = 0.841 at Time 2.

### 4.5. Data Analysis

Data analyses were conducted using SPSS 19.0 and AMOS 19.0. Data preprocessing was conducted first. Descriptive statistics were computed using all available data, and pairwise deletion was applied to the correlation matrix. For the structural equation model, missing data were handled using full information maximum likelihood estimation. Because neglect, violence, and growth mindset were assessed using adolescent self-reports, common method variance may have compromised the validity of the findings. Therefore, the Unmeasured Latent Method Factor (ULMF) approach was applied to examine this issue (Podsakoff et al., 2003).

Next, descriptive statistics and bivariate correlations were calculated. Guided by the proposed hypotheses, an initial two-wave cross-lagged panel model (CLPM) was estimated to examine the associations among parental burnout, parental neglect, parental violence, and adolescents’ growth mindset across the two waves (*n* = 382). Given the poor fit of the initial model, the nonsignificant paths were removed, and the shared variance between relevant variables was controlled. The initial model was then respecified to obtain the final model.

Finally, two supplementary analyses were conducted to examine the robustness of the findings. First, the final model was re-estimated using Bayesian estimation based on the same sample of 382 matched parent–adolescent dyads used in the cross-lagged panel model. Second, a supplementary structural equation modeling (SEM) with the maximum likelihood method was conducted using the 264 parent–adolescent dyads with complete data and no missing values across the two waves after listwise deletion.

## 5. Results

### 5.1. Missing Data Analysis

As some participants did not complete all questionnaires, Welch tests and chi-square tests were conducted to examine whether differences existed between the complete-response group and the incomplete-response group, thereby assessing whether missing data may have introduced bias. The results indicated no significant differences between the two groups in parental burnout (*t* = −1.554, *df* = 63.507, *p* = 0.125), violence (*t* = −0.415, *df* = 364, *p* = 0.679), growth mindset (*t* = −0.874, *df* = 361, *p* = 0.383), parent age (*t* = −0.631, *df* = 308, *p* = 0.529), adolescent age (*t* = −1.415, *df* = 367, *p* = 0.158), parent gender (χ^2^ = 0.470, *df* = 3, *p* = 0.925), parent education level (χ^2^ = 2.763, *df* = 2, *p* = 0.251), number of offspring (χ^2^ = 1.008, *df* = 2, *p* = 0.604), or adolescent gender (χ^2^ = 2.603, *df* = 2, *p* = 0.272). However, the two groups showed a marginally significant difference in neglect (*t* = −1.945, *df* = 152.055, *p* = 0.054), with the incomplete-response group (*M* = 1.181, *SD* = 1.025) reporting higher levels of neglect than the complete-response group (*M* = 0.963, *SD* = 0.793).

### 5.2. Common Method Bias Test

We conducted a series of confirmatory factor analyses to examine whether common method variance could bias our results. First, a measurement model was constructed for all measurement variables, with each item loading on its corresponding latent construct (Model 1). Second, a latent method factor that loaded on all items was added. The estimated variance of the method factor was 0.135, indicating that approximately 1.82% of the item variance was attributable to the common method factor. This proportion was lower than the 25% threshold for method variance (Williams et al., 1989), indicating that common method bias was relatively small in the present study.

#### Correlation Analysis

As shown in Table 1, the key correlations among the main study variables were significant and in the expected directions. T1 parental burnout was significantly and positively correlated with T2 parental burnout (*r* = 0.28, *p* < 0.01), T2 neglect (*r* = 0.29, *p* < 0.01), and T2 violence (*r* = 0.26, *p* < 0.01). T1 parental burnout was also significantly and negatively correlated with T1 growth mindset (*r* = −0.11, *p* < 0.05) and T2 growth mindset (*r* = −0.13, *p* < 0.05). In addition, T1 neglect was significantly and negatively correlated with T2 growth mindset (*r* = −0.30, *p* < 0.01), and T1 violence was significantly and negatively correlated with T2 growth mindset (*r* = −0.31, *p* < 0.01). These correlations provided preliminary evidence for the expected associations.

### 5.3. Cross-Lagged Panel Model Analysis

**Figure 1:**
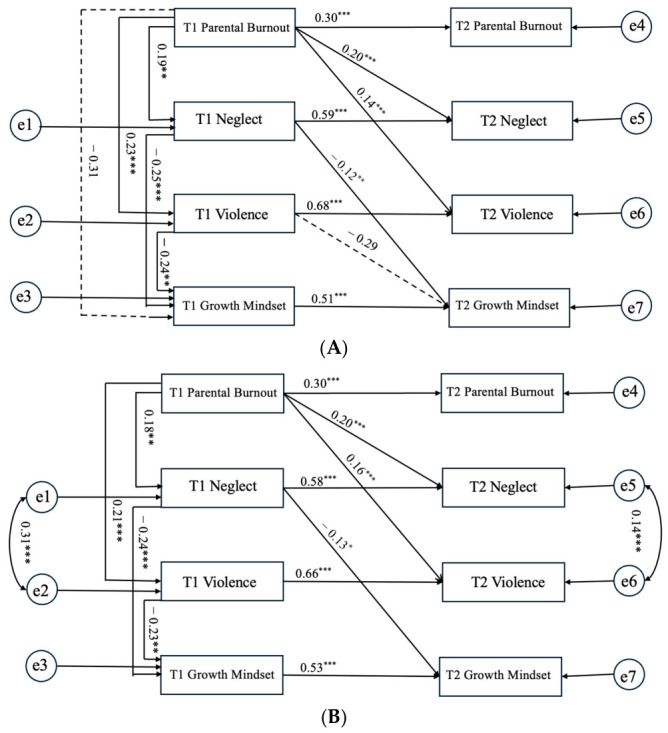
(**A**,**B**) T1 = Time 1; T2 = Time 2. * *p* < 0.05, ** *p* < 0.01, *** *p* < 0.001. Dashed lines indicate statistically non-significant associations.

Based on the proposed hypotheses, an initial model was first estimated, as shown in Figure 1A. The initial model showed poor goodness of fit, χ^2^(15) = 222.03, *p* < 0.001, RMSEA = 0.190, CFI = 0.778, and TLI = 0.586. Therefore, the model was modified with modification indices. Specifically, the paths from T1 parental burnout to T1 growth mindset (β = −0.31, *p* > 0.05) and from T1 parental violence to T2 growth mindset (β = −0.29, *p* > 0.05) were removed. In addition, residual covariances between parental neglect and parental violence were freely estimated at both Time 1 and Time 2, yielding the final model (Figure 1B).

The final model is presented in Figure 1B. The final exploratory path model showed acceptable fit, χ^2^(15) = 39.15, *p* < 0.001, RMSEA = 0.065, CFI = 0.978, and TLI = 0.952. After controlling for autoregressive paths from T1 to T2, T1 parental burnout was significantly and positively associated with T2 parental neglect (a-path; β = 0.20, *p* < 0.001), and T1 parental neglect was significantly and negatively associated with T2 growth mindset (b-path; β = −0.13, *p* < 0.01). The indirect association was statistically significant but small (indirect effect: a*b = −0.02, 95% CI [−0.033, −0.003], *p* < 0.05). The residual covariance between parental neglect and parental violence was significant at both Time 1 (*r* = 0.31, *p* < 0.001) and Time 2 (*r* = 0.14, *p* < 0.001).

### 5.4. Supplementary Analyses

To examine the robustness of the main findings, two supplementary analyses were conducted. First, the final exploratory path model was re-estimated using Bayesian estimation based on the same analytic sample. The Bayesian results were generally consistent with the main FIML results. Specifically, the posterior estimates indicated that T1 parental burnout was positively associated with T2 neglect (posterior mean = 0.173, 95% credible interval [0.094, 0.251], standardized estimate = 0.190) and T2 violence (posterior mean = 0.128, 95% credible interval [0.053, 0.203], standardized estimate = 0.143). T1 neglect was negatively associated with T2 growth mindset (posterior mean = −0.095, 95% credible interval [−0.165, −0.024], standardized estimate = −0.125). The 95% credible intervals for these paths did not include zero. The indirect pathway involving neglect was also small, with a 95% credible interval that did not include zero (posterior mean = −0.016, 95% credible interval [−0.031, −0.002], standardized estimate = −0.024).

Second, a supplementary maximum likelihood analysis was conducted using the 264 dyads with complete data across the two waves. The results were generally consistent with the main analysis. The final model showed acceptable fit, χ^2^(15) = 61.73, *p* < 0.001; RMSEA = 0.070; CFI = 0.980; TLI = 0.950; SRMR = 0.060. After controlling for the autoregressive paths between T1 and T2, T1 parental burnout was positively associated with T2 neglect (β = 0.22, *p* < 0.001), and T1 neglect was negatively associated with T2 growth mindset (β = −0.13, *p* < 0.01). Bootstrapping analysis with 5000 samples showed that the indirect effect was significant but small (indirect effect = −0.02, 95% CI [−0.042, −0.002], *p* < 0.05).

## 6. Discussion

This study explored the associations among parental burnout, parental neglect, parental violence, and adolescents’ growth mindset using two-wave parent–adolescent dyadic data. The findings provide preliminary evidence that parental burnout was negatively correlated with adolescents’ growth mindset and was positively associated with subsequent parental neglect and parental violence; parental neglect may serve as an important linking pathway between parental burnout and lower levels of adolescents’ growth mindset. During the exploratory model respecification process, two nonsignificant paths were removed: one from T1 parental burnout to T1 growth mindset and the other from T1 parental violence to T2 growth mindset. Parental burnout was conceptualized as a relatively distal family risk factor that may be associated with adolescents’ growth mindset through parenting behaviors and parent–adolescent interactions. In addition, because parental neglect and parental violence may co-occur within the same family context and be influenced by common underlying factors (Debowska et al., 2017), residual covariances between the two constructs were freely estimated at both time points. Because the final model included only two time points, was derived through exploratory respecification based on the fit indices, and yielded a small indirect effect, the findings should be interpreted cautiously.

Parental burnout and adolescents’ growth mindset were negatively correlated in the correlation analysis. However, this association was not statistically significant in the structural equation model, suggesting that this relation may not be stable or the relation may be mediated by other variables. The emotional exhaustion and psychological distancing associated with parental burnout (Roskam et al., 2018) may not directly influence adolescents’ endorsement of the belief that abilities can be improved through effort. Meanwhile, parental burnout may be more likely to be associated with adolescents’ growth mindset through changes in parents’ everyday parenting behaviors and parent–adolescent interactions.

The development of a growth mindset relies on continuous parental feedback and cognitive support (W. Peng & Zhang, 2025). Previous research has shown that when parents provide insufficient emotional responsiveness, adolescents may receive less positive feedback regarding their learning and problem-solving efforts, which may in turn hinder learning outcomes (Brooker & Poulin-Dubois, 2013). This lack of reinforcement may make it more difficult for adolescents to form cognitive links between effort and ability improvement, thereby hindering the development of a growth mindset. In addition, studies have found a significant negative association between early adversity and lower levels of growth mindset (Lurie et al., 2023). As a form of early adversity, neglect may deprive adolescents of the cognitive and emotional support and feedback they would otherwise receive during learning and exploration (McLaughlin et al., 2014). However, this finding was derived only from an exploratory model based on two-wave data, and the indirect effect was small. Therefore, this finding requires further examination in future research.

Further, parental violence did not emerge as a significant indirect pathway linking parental burnout to adolescents’ growth mindset. Parental burnout was positively associated with later violence. Parental burnout is typically characterized by emotional exhaustion, emotional distancing, and a reduced sense of fulfillment in parenting (Roskam et al., 2018), which may make parents more likely to respond with anger or corporal punishment when facing daily parenting stress or parent–adolescent conflicts. Violence was not retained as a pathway to growth mindset in the final model. The development of a growth mindset depends largely on individuals’ long-term beliefs about their abilities, learning experiences, and positive feedback (L. Peng et al., 2025). Parental violence or corporal punishment can negatively affect adolescents’ emotional security (Afifi et al., 2006; Tomoda et al., 2009), leading to outcomes such as anxiety and depression. Disciplinary practices may be interpreted differently across cultural contexts. The concept of cultural normativeness suggests that when certain disciplinary practices are more common within a cultural context, adolescents may be more likely to perceive them as normative rather than purely hostile behaviors. However, research also indicates that cultural normativeness does not eliminate the potential harmful consequences of such practices (Lansford et al., 2005). Therefore, the nonsignificant indirect pathway involving violence in the present study may reflect the complex psychological processes through which adolescents interpret and internalize parental disciplinary behaviors. Future research should further examine how contextual and cultural factors shape the developmental consequences of parental violence for adolescents.

### 6.1. Theoretical and Practical Implications

Building on existing research, this study provides preliminary evidence for understanding the relationship between parental burnout and adolescents’ growth mindset. It further enriches the literature on the potential consequences of parental burnout and offers a basis for exploring the links between parents’ psychological states and adolescents’ growth-related factors. The findings suggest that parental burnout may be associated with lower levels of adolescents’ growth mindset through parental neglect. Compared with parental violence, parental neglect may play a more salient linking role between parental burnout and adolescents’ growth mindset, providing preliminary evidence for understanding the relationships among parents’ psychological states, negative parenting behaviors, and adolescent development. From a practical perspective, the present findings suggest that parental burnout and daily parent–adolescent interactions may be relevant to adolescents’ growth mindset. Parents should recognize that, compared with parental violence, neglect in everyday parent–adolescent interactions may be more closely related to adolescents’ development. In addition, individuals with higher levels of neglect exhibited a higher rate of missing responses, suggesting that those experiencing greater neglect may be more likely to withdraw from the study. Although this difference was only marginally significant, it further indicates, to some extent, the detrimental effect of neglect on offspring. Parents should therefore pay greater attention to the potential effects of their own neglectful behaviors on adolescents.

### 6.2. Limitations and Directions for Future Research

Although this study provided preliminary evidence regarding the associations among parental burnout, neglect, violence, and adolescents’ growth mindset, several limitations should be noted.

First, this study used a two-wave semi-longitudinal design with a one-month interval. Although measuring all variables at both time points allowed us to control for the autoregressive path and examine short-term temporal associations, this design was still insufficient to provide adequate temporal evidence for a complete mediation process and could not distinguish within-family changes from stable between-family differences. In addition, the final model was obtained through exploratory modifications after the initially hypothesized model showed poor fit, a procedure that may increase the risk of Type I error. Future research should collect data across at least three waves, use longer intervals between assessments, and consider random-intercept cross-lagged panel models to examine within-family developmental processes more rigorously (Kamza et al., 2026).

Second, parental burnout was assessed using the total score of the seven-item short version of the Parental Burnout Assessment. Although this measure showed good internal consistency in the present sample, it did not allow us to examine the specific roles of different parental burnout symptoms. Future research should use the full Parental Burnout Assessment and adopt symptom-level analytic approaches (Carone et al., 2026) to examine whether different parental burnout symptoms are differentially associated with neglect, violence, and adolescents’ developmental outcomes.

Third, this study focused on only one aspect of growth-related factors, namely growth mindset, without considering other developmental resources, such as psychological resilience, emotional intelligence, and external support (Masten & Barnes, 2018; Megías-Robles et al., 2024; Scales, 2014). Future research could expand the range of growth-related indicators to provide a more comprehensive assessment of adolescents’ developmental resources.

## 7. Conclusions

Using matched parent–adolescent dyadic data, a two-wave semi-longitudinal design, and an exploratory cross-lagged panel model, this study examined the relationships among parental burnout, parental neglect, parental violence, and adolescents’ growth mindset. The results suggest that parental neglect may be important for understanding the link between parental burnout and lower levels of growth mindset among adolescents.

## Figures and Tables

**Table 1:** Descriptive Statistics and Correlation Analysis.

Variable	*M* (*SD*)	1	2	3	4	5	6	7	8	9	10	11	12	13
1 PG	1.75 (0.54)													
2 PA	40.84 (6.54)	−0.06												
3 PEL	1.29 (0.47)	−0.08	−0.03											
4 NO	1.94 (0.66)	0.10	−0.05	−0.2 **										
5 AG	1.46 (0.51)	0.07	0.03	−0.07	0.17 **									
6 AA	13.04 (0.74)	−0.02	0.07	−0.06	0.02	−0.10								
7 T1PB	1.53 (0.93)	0.08	−0.01	−0.03	0.02	0.09	0.08							
8 T2PB	1.49 (0.91)	0.08	0.03	−0.01	−0.03	−0.02	0.09	0.28 **						
9 T1NE	1.02 (0.87)	0.11	0.07	−0.05	−0.04	0.04	0.16 **	0.17 **	0.13 *					
10 T2NE	0.96 (0.87)	0.08	0.10	−0.01	0.04	0.06	0.23 **	0.29 **	0.19 **	0.64 **				
11 T1VI	1.76 (0.76)	0.10	0.05	0.08	−0.05	0.03	0.15 **	0.20 **	0.19 **	0.51 **	0.56 **			
12 T2VI	1.77 (0.87)	0.05	0.08	0.02	−0.04	0.07	0.14 **	0.26 **	0.16 **	0.41 **	0.62 **	0.72 **		
13 T1GM	3.30 (0.55)	−0.11	0.04	−0.04	−0.01	−0.15 **	−0.08	−0.11 *	−0.09	−0.36 **	−0.34 **	−0.37 **	−0.27 **	
14 T2GM	3.31 (0.66)	−0.02	−0.12 *	0.06	0.01	−0.18 **	−0.09	−0.13 *	−0.02	−0.30 **	−0.38 **	−0.31 **	−0.35 **	0.57 **

Note. PG = Parent Gender; PA = Parent Age; PEL = Parent Education Level; NO = Number of Offspring; AG = Adolescent Gender; AA = Adolescent Age; PB = Parental Burnout; NE = Neglect; VI = Violence; GM = Growth Mindset; T1 = Time 1; T2 = Time 2. * *p* < 0.05, ** *p* < 0.01. Means, standard deviations, and correlations were calculated using all available data. Pairwise deletion was used for bivariate correlations; therefore, sample sizes varied across variables and correlations.

## Data Availability

The data presented in this study are available on request from the corresponding author due to privacy restrictions.
